# Soil-Transmitted Helminth Reinfection after Drug Treatment: A Systematic Review and Meta-Analysis

**DOI:** 10.1371/journal.pntd.0001621

**Published:** 2012-05-08

**Authors:** Tie-Wu Jia, Sara Melville, Jürg Utzinger, Charles H. King, Xiao-Nong Zhou

**Affiliations:** 1 Key Laboratory on Biology of Parasites and Vectors, MOH, WHO Collaborating Center on Malaria, Schistosomiasis and Filariasis, National Institute of Parasitic Diseases, Chinese Center for Disease Control and Prevention, Shanghai, People's Republic of China; 2 Hughes Hall College, Cambridge University, Cambridge, United Kingdom; 3 Department of Epidemiology and Public Health, Swiss Tropical and Public Health Institute, Basel, Switzerland; 4 University of Basel, Basel, Switzerland; 5 Center for Global Health and Diseases, Case Western Reserve University School of Medicine, Cleveland, Ohio, United States of America; Universidad San Francisco de Quito, Ecuador

## Abstract

**Background:**

Soil-transmitted helminth (STH) infections (i.e., *Ascaris lumbricoides*, hookworm, and *Trichuris trichiura*) affect more than a billion people. Preventive chemotherapy (i.e., repeated administration of anthelmintic drugs to at-risk populations), is the mainstay of control. This strategy, however, does not prevent reinfection. We performed a systematic review and meta-analysis to assess patterns and dynamics of STH reinfection after drug treatment.

**Methodology:**

We systematically searched PubMed, ISI Web of Science, EMBASE, Cochrane Database of Systematic Reviews, China National Knowledge Infrastructure, WanFang Database, Chinese Scientific Journal Database, and Google Scholar. Information on study year, country, sample size, age of participants, diagnostic method, drug administration strategy, prevalence and intensity of infection pre- and posttreatment, cure and egg reduction rate, evaluation period posttreatment, and adherence was extracted. Pooled risk ratios from random-effects models were used to assess the risk of STH reinfection after treatment. Our protocol is available on PROSPERO, registration number: CRD42011001678.

**Principal Findings:**

From 154 studies identified, 51 were included and 24 provided STH infection rates pre- and posttreatment, whereas 42 reported determinants of predisposition to reinfection. At 3, 6, and 12 months posttreatment, *A. lumbricoides* prevalence reached 26% (95% confidence interval (CI): 16–43%), 68% (95% CI: 60–76%) and 94% (95% CI: 88–100%) of pretreatment levels, respectively. For *T. trichiura*, respective reinfection prevalence were 36% (95% CI: 28–47%), 67% (95% CI: 42–100%), and 82% (95% CI: 62–100%), and for hookworm, 30% (95% CI: 26–34%), 55% (95% CI: 34–87%), and 57% (95% CI: 49–67%). Prevalence and intensity of reinfection were positively correlated with pretreatment infection status.

**Conclusion:**

STH reinfections occur rapidly after treatment, particularly for *A. lumbricoides* and *T. trichiura*. Hence, there is a need for frequent anthelmintic drug administrations to maximize the benefit of preventive chemotherapy. Integrated control approaches emphasizing health education and environmental sanitation are needed to interrupt transmission of STH.

## Author Summary

Infections with soil-transmitted helminths (the roundworm *Ascaris lumbricoides*, the whipworm *Trichuris trichiura*, and hookworm) affect over 1 billion people, particularly rural communities in the developing world. The global strategy to control soil-transmitted helminth infections is ‘preventive chemotherapy’, which means large-scale administration of anthelmintic drugs to at-risk populations. However, because reinfection occurs after treatment, ‘preventive chemotherapy’ must be repeated regularly. Our systematic review and meta-analysis found that at 3, 6, and 12 months after treatment, *A. lumbricoides* prevalence reached 26% (95% confidence interval (CI): 16–43%), 68% (95% CI: 60–76%) and 94% (95% CI: 88–100%) of pretreatment levels, respectively. For *T. trichiura*, respective reinfection prevalence at these time points were 36% (95% CI: 28–47%), 67% (95% CI: 42–100%), and 82% (95% CI: 62–100%); and for hookworm, 30% (95% CI: 26–34%), 55% (95% CI: 34–87%), and 57% (95% CI: 49–67%). Prevalence and intensity of reinfection were positively correlated with pretreatment infection status. Our results suggest a frequent anthelmintic drug administration to maximize the benefit of preventive chemotherapy. Moreover, an integrated control strategy, consisting of preventive chemotherapy combined with health education and environmental sanitation is needed to interrupt transmission of soil-transmitted helminths.

## Introduction

Infections with soil-transmitted helminths (STHs) affect more than 1 billion people, particularly the rural poor of the developing world [1], [2]. The four most common STHs are the roundworm (*Ascaris lumbricoides*), the whipworm (*Trichuris trichiura*), and two hookworm species (*Ancylostoma duodenale* and *Necator americanus*) [2]. The greatest number of STH infections occur in Central and South America, People's Republic of China (P.R. China), Southeast Asia, and sub-Saharan Africa [2], [3]. Warm climates and adequate moisture are essential for the hatching or embryonation of STH eggs in the environment or development of larvae.

Important contextual determinants for human infection are poverty, lack of sanitation, and inadequate hygiene (e.g., absence of hand washing with soap after defecation and before eating, and walking barefoot) [4]–[6]. In such social-ecological systems, multiple species STH infections are common [7].

Transmission of STHs occurs via contact with contaminated soil (hookworm) or consumption of egg-contaminated foods (*A. lumbricoides* and *T. trichiura*) [4]. An important epidemiological feature is their highly aggregated distribution: the majority of patients harbor low intensity infections, while only few individuals harbor very heavy infections [8]. People infected with STHs may suffer from anemia, growth stunting, diminished physical fitness, and impaired cognitive development [7], representing a persistent drain on social and economic development of low-income countries [9], [10]. The current global strategy to control STH infections is preventive chemotherapy, that is the repeated large-scale administration of anthelmintic drugs to at-risk populations, most importantly school-aged children [11], [12]. A shortcoming of this strategy is failure to prevent reinfection after effective deworming [6], [7], [13], [14]. Hence, identifying factors that determine reinfection risk is crucial to improving the effectiveness of this strategy [15].

To foster the design of more effective integrated control strategies, the objectives of this systematic review and meta-analysis were to assess available evidence on global patterns of STH reinfection after drug treatment, and to identify, through pooled risk estimates, the frequency and leading determinants of STH reinfection.

## Methods

This systematic review was developed in line with the PRISMA guidelines (see Checklist S1) [16]. A protocol was prospectively registered in PROSPERO [17], registration number: CRD42011001678; available from http://www.crd.york.ac.uk/PROSPERO/display_record.asp?ID=CRD42011001678.

### Selection Criteria

**Figure 1:**
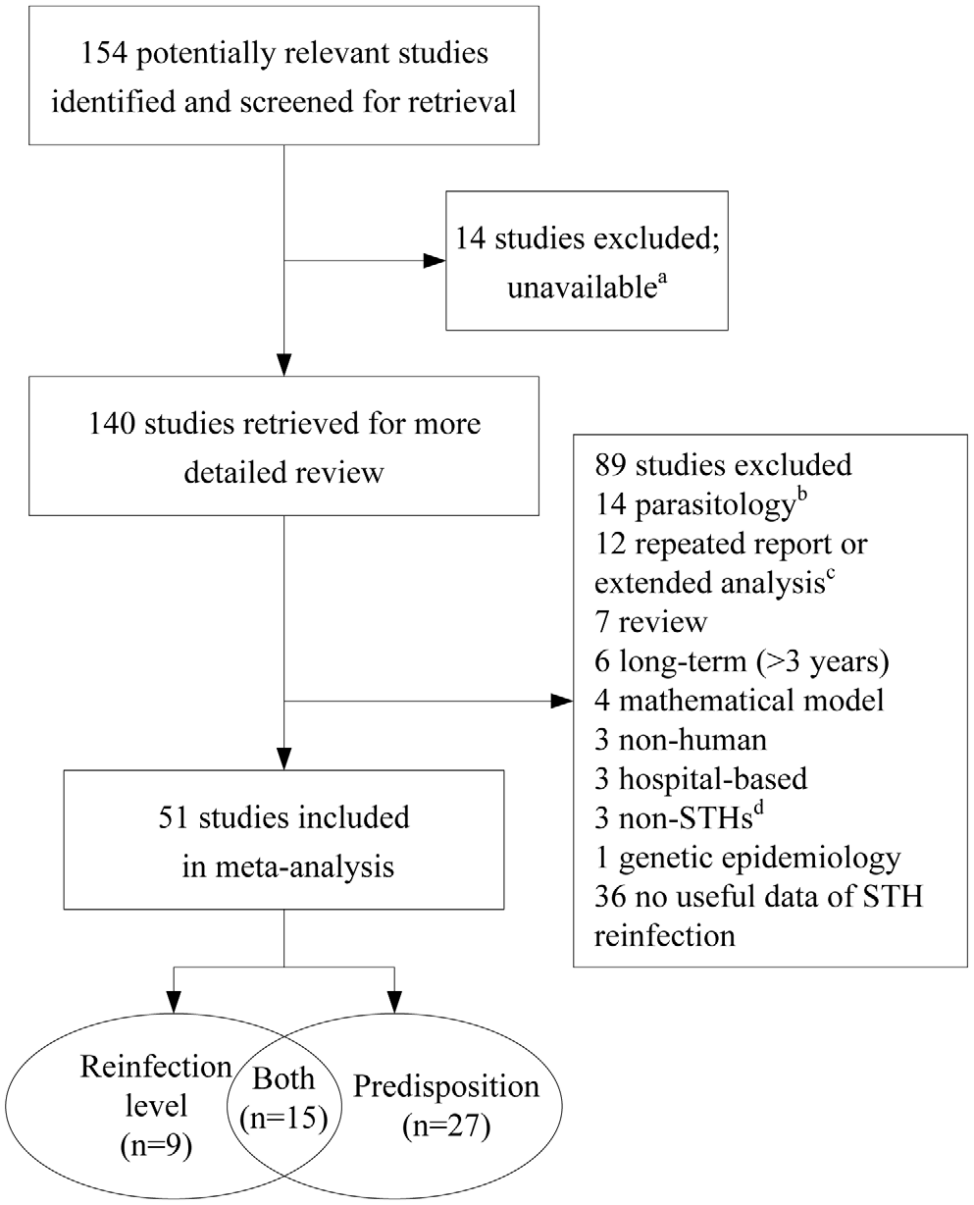
Flowchart visualizing the procedure for identifying relevant publications. ^a^The original article or full text could not be obtained. ^b^14 parasitology: 9 dynamics and structure of population, 4 immunology, 1 quantitative measure method of reinfection. ^c^Extended analysis of previously published study. ^d^2 on enteroparasite (intestinal parasites including *Enterobius vermicularis*, *Hymenolepis nana*, *Giardia intestinalis*, *Entamoeba coli*, *Entamoeba histolytica*, and others), 1 on bancroftian filariasis.

We aimed to include all published studies in English or Chinese in which reinfection with STH was measured, for the period January 1, 1900 to December 31, 2010. Both observational studies and trials were eligible for inclusion. We excluded the following studies: (i) data without rate of infection after preventive chemotherapy; (ii) where time of follow-up was less than 2 months or more than 3 years; (iii) hospital-based or case studies in which the representativeness of the sample for the general population was unknown; and (iv) duplicate publication or extended analysis of previously published studies (see Figure 1 for selection flow of included studies). Additional exclusion criteria were: low adherence (loss rate of subjects at follow-up >30%), low initial prevalence (<10%), and poor cure rate (CR; i.e., <80% for *A. lumbricoides*, <20% for *T. trichiura*, or <20% for hookworm) unless cure was boosted by repeated treatment [18]. In our review, CR is defined as the percentage of STH-positive individuals who became egg-negative posttreatment, as assessed within 7 to 60 days after treatment [18], [19]. Adherence was defined as the percentage of individuals who were followed-up, and hence included in a per-protocol analysis.

### Search Strategy

We identified published studies using the National Library of Medicine's PubMed (1900 to December 2010), ISI Web of Science (1900 to December 2010), EMBASE (1947 to December 2010), China National Knowledge Infrastructure (CNKI; 1979 to December 2010), WanFang Database (1980 to December 2010), and Chinese Scientific Journal Database (VIP; 1989 to December 2010). We employed the following terms (phoneticism (Pinyin) of Chinese keywords given in parenthesis): *helminth* (*ruchong*), or *nematode* (*xianchong*), or *Ascaris* (*huichong*), or *hookworm* (*gouchong*), or *whipworm*, or *Trichuris* (*bianchong*) and *reinfection* (*zaiganran*), or *chemotherapy* (*hualiao*), or *albendazole* (*abendazuo* or *bingliumizuo*), or *mebendazole* (*jiabendazuo*), or *levamisole* (*zuoxuanmizuo*), or *pyrantel pamoate* (*saimiding*) and *longitudinal study* (*zongxiang*), or *cohort study* (*duilie*), or *trial* (*shiyan*).

Automated database searches covered the period from when earliest electronic records were available (e.g., 1900 in the case of ISI Web of Science) to the end of December 2010. Additional studies published before and during this period were identified by hand-searching reference lists of obtained articles or published abstracts of older literature from the Cochrane Database of Systematic Reviews (CDSR), and by searching Google Scholar using the terms *helminth* and *reinfection*. Identified reports were imported into EndNote X2 (Thomson Reuters) for management. Two experienced, independent reviewers (TWJ, SM) assessed studies against inclusion and exclusion criteria according to a pre-established study protocol. Tables S1 and S2 give details of 139 retrievable studies recovered by our search, which were ultimately included (n = 51, Table S1) or excluded in our meta-analyses (n = 88, Table S2).

### Outcome Assessments

We abstracted and compared the following components associated with STH reinfection: rate and intensity of infection before and after treatment, CR, time between evaluations before and after treatment, and adherence to follow-up. Additional study characteristics included year and country where the trial was implemented, sample size, age of study participants, diagnostic method and number of stool samples examined, and strategy of drug administration (universal, targeted, or selective treatment).

In the first phase of assessment, outcomes related to STH reinfection of different cohorts or subgroups were analyzed separately, and only one cohort was included per study. Those cohorts without statistically significant differences in infection rates both before and after treatment were combined and analyzed as one, but those with significant differences were selected according to adherence rate first, then CR or initial prevalence. If multiple rounds of chemotherapy were administered within 1 year, expecting a relatively higher CR, we conservatively selected the prevalence after the latest treatment as the main outcome. Finally, data were stratified by species of STH, subgroup, and time interval of follow-up after treatment, categorized as ‘3 months’ (3–4 months), ‘6 months’ (6–8 months) or ‘12 months’ (10–12 months).

### Data Analysis

Review Manager software 5.0.23, provided by the Cochrane Collaboration group, was used for meta-analysis. Risk ratios (RR) were listed by study and compared for the risk or predisposition to reinfection after preventive chemotherapy. The primary analysis was performed according to helminth species-specific infection and length of follow-up intervals. Additional analysis was performed for specific population subgroups. Prevalence risk ratio (PRR) was utilized as indicator for estimating the risk of reinfection after treatment according to the following formula: 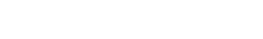 [20]. Hence, PRR reflects the re-acquired level of infection after treatment, in comparison with the pretreatment level. In some studies, this indicator was directly used as the reinfection rate [21], [22].

Subgroup analysis was based on initial infection status, age, sex, and selected socioeconomic factors (Table 1). We calculated pooled estimates with a random-effects model, unless heterogeneity level determined by Moran's *I* ^2^ was less than 50% [23]. Of note, *I* ^2^ represents the proportion of the between-study variance of the total variance in a pooled estimate. An *I* ^2^ of 0% indicates that all variability in effect estimates is due to sampling error within studies, and that none is due to heterogeneity [24], [25]. Publication bias was visually examined using funnel plots [26]. Results are presented by means of forest plots, including 95% confidence intervals (CI). In the figures and tables presented, RR not equal to 1 indicates that reinfection rate is associated with the key variable underlying the subgroup analysis (e.g., age and sex).

**Table 1:** Summary of 51 included studies reporting on posttreatment reinfection by soil-transmitted helminths (STHs), stratified by parasite species.

	Number of studies by species^*^			
	*A. lumbricoides*	*T. trichiura*	Hookworm	Number of studies [reference(s)]
*1. Prevalence of STH infection after treatment*	21	12	14	24 [13], [19], [22], [27]–[47]
*2. Predisposition factors*				
Initial infection (present or absent)	8	5	3	9 [31], [44], [48]–[54]
Initial infection intensity (light or heavy)	9	8	6	16 [32], [35], [49], [50], [52], [53], [55]–[64]
Age	8	5	5	11 [32]–[34], [44], [49], [51], [54], [57], [62], [65], [66]
Sex	8	4	4	9 [21], [32]–[34], [44], [51], [54], [65], [67]
*3. Other factors*				
Nutrient supplementation	4	3	3	4 [19], [22], [68], [69]
Malnutrition or growth-retarded	2	2	1	2 [21], [39]
Health promotion	2	2	2	2 [37], [70]
Hand washing with soap	1	0	0	1 [71]
Geophagy	1	1	1	1 [72]
Education or occupation of mother	2	1	1	2 [21], [50]
Crowded housing	2	0	0	2 [50], [64]
Type of floor or yard	3	1	0	3 [13], [61], [65]
Sanitary latrine	3	1	3	4 [21], [27], [50], [73]
Vegetable plantation area	1	0	1	1 [41]
Seasonal fluctuation	3	0	0	3 [13], [29], [48]
Total	43	27	27	51

## Results

### Studies Identified and Characteristics

Of the 154 studies identified through our systematic database searches, complemented with hand searches, 51 studies carried out in 26 countries met inclusion criteria for subsequent meta-analysis (Figure 1). Twenty-four studies presented STH infection rates of populations or mixed cohorts, including information on the numbers infected and uninfected before and after treatment that could be used to assess the re-acquired prevalence of STH infections at specified time intervals posttreatment. Forty-two studies provided reinfection data by subgroups and evaluated their relative predisposition to reinfection. Table 1 summarizes the number of studies related to reinfection and its potential determinants, stratified by species of STH.

Of the 51 studies included, 84% provided data on *A. lumbricoides* after treatment, 53% on *T. trichiura*, and 53% on hookworm. Twenty-five studies investigated single-species infection after treatment: 17 for *A. lumbricoides* alone, five for hookworm alone, and three for *T. trichiura* alone, 20 assessed all three STH species concurrently, four assessed *A. lumbricoides* and *T. trichiura*, and two assessed *A. lumbricoides* and hookworm.

### Dynamics of Reinfection

Twenty-four studies provided STH infection rates before and after treatment, including sample sizes. Most of these focused on *A. lumbricoides* and on reinfection in the 6–12 months follow-up period (Table S3). We performed nine pooled estimates (3 species×3 time points) of reinfection level in total, but were unable to pool the RRs of *T. trichiura* and hookworm at 3 months after treatment due to an insufficient number of studies. Heterogeneity was high for all pooled estimates (*I* ^2^>80%) [24], even after exclusion of five studies due to poor quality (three with low adherence rates [32], [36], [39], two with poor CR [13], [28]; for details, see Table S3). Publication and selection bias were not detected visually using funnel plots in eight pooled estimates with the exception of *A. lumbricoides* at the 3 months posttreatment follow-up time point.

### Re-Acquired Prevalence of STH at 3 Months Posttreatment

**Figure 2:**
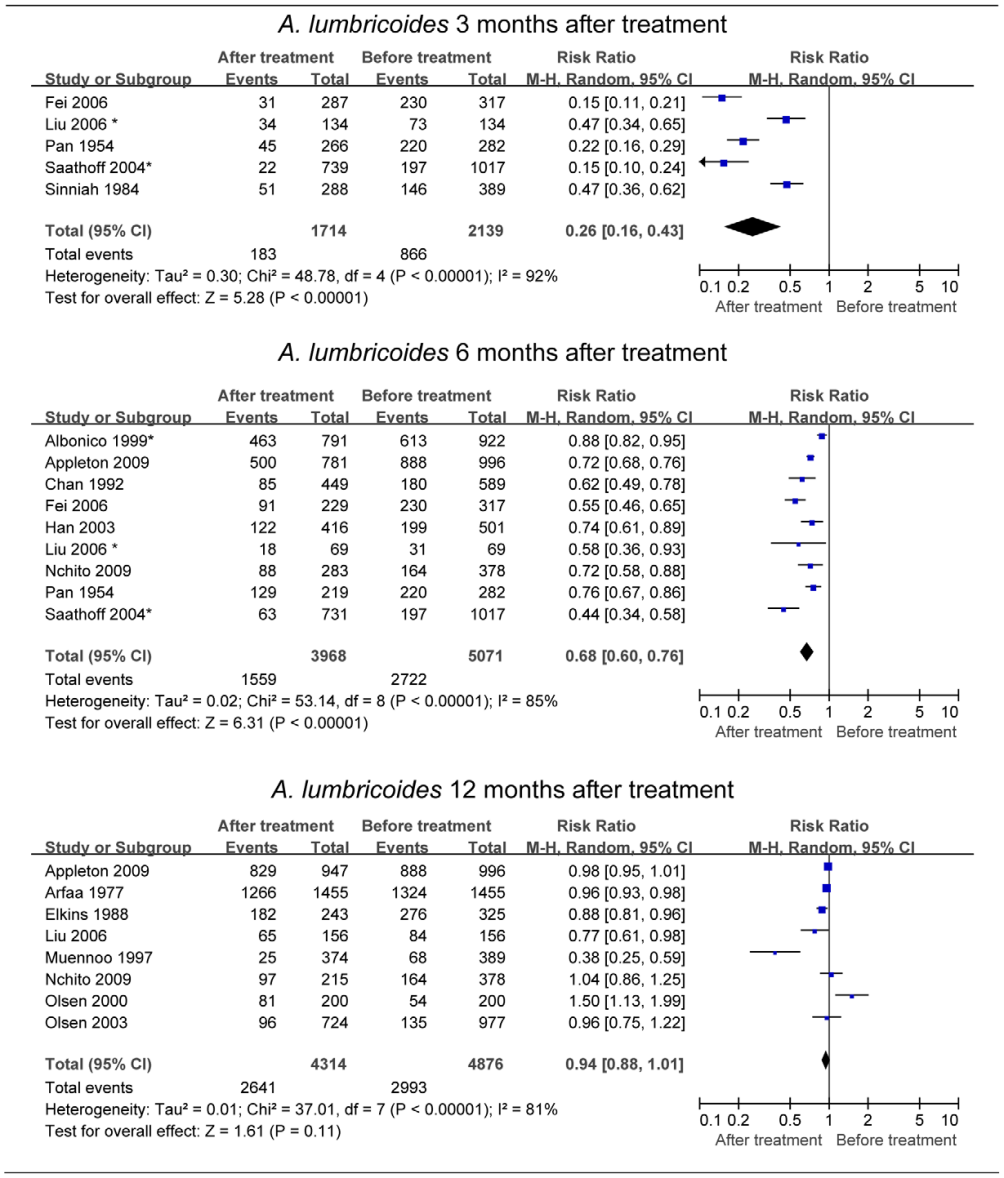
Forest plot of prevalence of *Ascaris lumbricoides* 3, 6, and 12 months posttreatment. A random relative risk (RR) value of less than 1 indicates a lower infection rate after treatment compared to the initial level. Diamonds represent the pooled estimate across studies. See Table S1 for full references. *The infection rate 3 or 6 months after the last round of treatment was abstracted (Table S3).

For *A. lumbricoides*, we found a random pooled RR of reinfection, based on five studies, of 0.26 (95% CI: 0.16–0.43) [29], [31], [42]–[44]. The estimates from the individual studies are listed in Figure 2. CRs ranged from 89.7% to 97.7% for three studies [31], [42], [43]. The remaining two studies applied an excluding or retreatment procedure to the uncured [29], [44]. The study carried out by Liu *et al.* (2006) [44] showed that even after four rounds of treatment with pyrantel pamoate (10 mg/kg per dose given at 3 months interval for a year), the prevalence of *A. lumbricoides* reached 47% of the original prevalence at 3 months after the final treatment. We also performed a subgroup analysis of school-aged children (6–15 years) after excluding one population-based study [44]. Their pooled RR from four studies was 0.22 (95% CI: 0.13–0.39) [29], [31], [42], [43], slightly less than the value obtained using all age groups. We performed another pooled estimate in view of the detected publication bias caused by one large study (n = 1017) [42]. After excluding this study, the pooled RR changed slightly, rising from 0.26 to 0.29 (95% CI: 0.17–0.51) [29], [31], [43], [44].

Due to low CR, only a few studies were included in our evaluation of reinfection with *T. trichiura* or hookworm at the 3 months posttreatment follow-up. In this review only two studies of *T. trichiura* and hookworm were included, which is insufficient for pooling [31], [42]. If the threshold of CR was set at 50%, then the resulting RR 3 months after treatment was 0.36 (95% CI: 0.28–0.47) for *T. trichiura* [31], and 0.30 (95% CI: 0.26–0.34) for hookworm (Table S3) [42].

### Re-Acquired Prevalence of STH 6 Months Posttreatment

For *A. lumbricoides*, the random pooled RR derived from nine studies was 0.68 (95% CI: 0.60–0.76) [29], [34], [40]–[44], [46], [47]. Estimates of the individual studies are given in Figure 2. CRs ranged from 92% to 100% in four studies [34], [40], [41], [43], while the remaining five studies applied an excluding or retreatment procedure to the uncured, and hence resulted in a higher CR [29], [42], [44], [46], [47]. We also performed a subgroup analysis of children aged 2–15 years after excluding two population-based studies and obtained a pooled estimate of 0.69 (95% CI: 0.60–0.79) [34], [44]. After three outlier studies were removed [40], [42], [43], the fixed RR was estimated to be 0.71 (95% CI: 0.68–0.75) (*I* ^2^ = 0%; χ^2^ = 3.22, *P* = 0.67) [29], [34], [41], [44], [46], [47].

**Figure 3:**
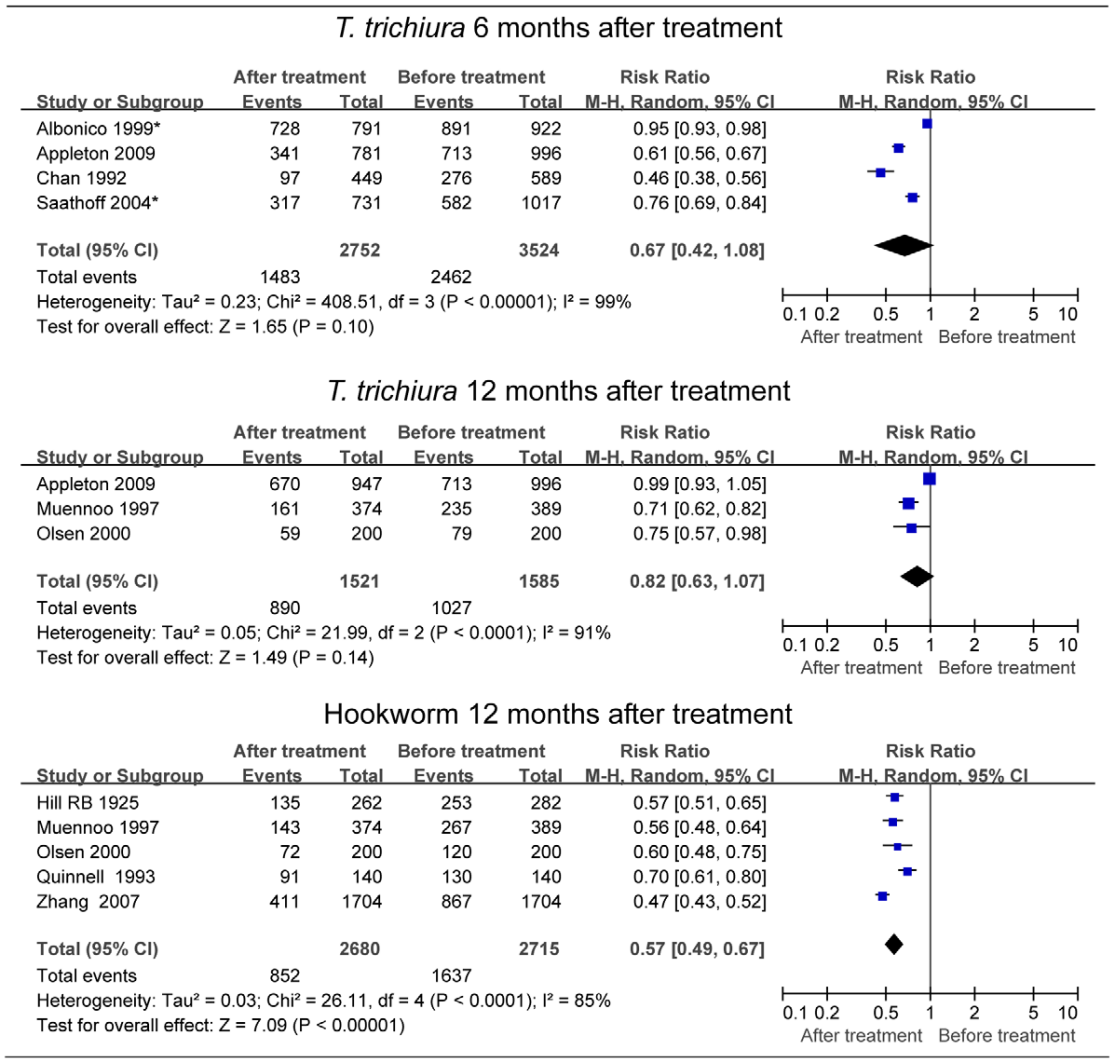
Forest plot of prevalence of *Trichuris trichiura* or hookworm after treatment. A random relative risk (RR) value of less than 1 indicates a lower infection rate after treatment compared to the initial level. Diamonds represent the pooled estimate across studies. See Table S1 for full references. *The infection rate 6 months after the last round of treatment was abstracted (Table S3).

For *T. trichiura*, four studies were included in the pooled estimate, two with moderate CRs (>67%) [34], [47], and two with poor CRs (30–40%), even after two rounds of treatment at 6-month intervals in 1 year (Table S3) [40], [42]. Poor CRs caused two outlier results and a higher pooled estimate without a significant difference from 1 (RR = 0.67; 95% CI: 0.42–1.08) (Figure 3) [34], [40], [42], [47]. When these two outlier studies were excluded [40], [42], the random RR dropped to 0.54 (95% CI: 0.41–0.71) [34], [47]. For hookworm, there were only two studies included with high CR (>90%) [41], [42], and the random RR was 0.55 (95% CI: 0.34–0.87).

### Re-Acquired Prevalence of STH 12 Months Posttreatment

Generally, the heterogeneity effect caused by CR, treatment regimen, subpopulation, and variability in study design decreased gradually over the posttreatment follow-up time and became non-significant. STH prevalence tended to regress to the pretreatment level in *A. lumbricoides* and *T. trichiura*, and persisted at approximately half the level in the case of hookworm. However, poor adherence in some studies was a major source of heterogeneity, which could introduce an influential outlier. This effect was evaluated for a study in which the adherence rate for *A. lumbricoides* was relatively high (74%; 724/977), but considerably lower for *T. trichiura* (56%; 548/977) and hookworm (60%; 588/977) (Table S3) [22].

For *A. lumbricoides*, eight studies were included, resulting in a random RR of 0.94 (95% CI: 0.88–1.01) (Figure 2 and Table S3) [19], [22], [30], [33], [37], [44], [46], [47]. Removal of two outlier studies [19], [37], resulted in a fixed RR of 0.96 (95% CI: 0.93–0.98) (*I* ^2^ = 49%; χ^2^ = 9.85, *P* = 0.08).

For *T. trichiura*, there were three studies included with a random RR of 0.82 (95% CI: 0.62–1.07) (Figure 3 and Table S3) [19], [37], [47]. One study was excluded from this pooling due to poor adherence of the *T. trichiura* cohort (56%; 548/977), of which the individual RR was 0.42 (95% CI: 0.35–0.51) [22]. If combined into the random-effects model, the pooled estimate dropped to 0.69 (95% CI: 0.45–1.05). We ultimately removed this cohort of *T. trichiura* from the pooled estimate based on our exclusion criteria (Table S3) [22].

For hookworm, there were five studies included with a random RR of 0.57 (95% CI: 0.49–0.67) (Figure 3 and Table S3) [19], [27], [35], [37], [45]. After removing two outlier studies [35], [45], we obtained a fixed RR of 0.57 (95% CI: 0.52–0.62) from three studies (*I* ^2^ = 0%; χ^2^ = 0.32, *P* = 0.85) [19], [27], [37]. In contrast to *A. lumbricoides* and *T. trichiura*, the re-acquired prevalence of hookworm returned to only about half of the initial level. We also removed a cohort of hookworm from the pooled estimate due to poor adherence (Table S3) [22]. Including that study would have resulted in a slightly lower pooled estimate (RR = 0.53; 95% CI: 0.44–0.64).

**Figure 4:**
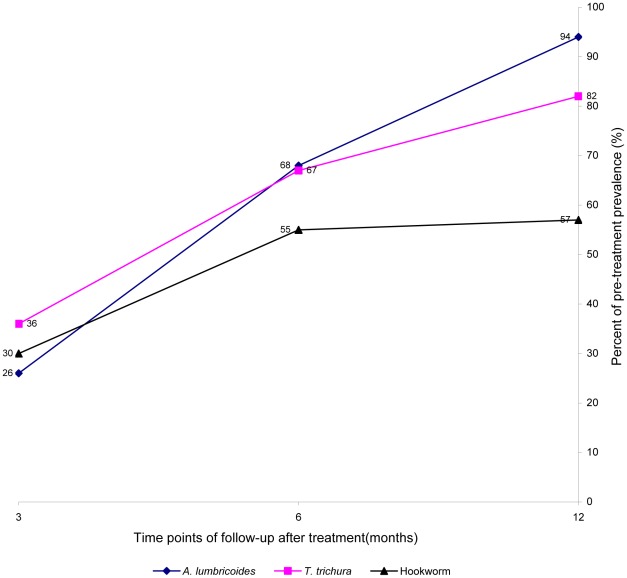
Summary of the rapidity of re-acquiring soil-transmitted helminth (STH) infections after treatment.

Figure 4 shows the rapidity of re-acquiring soil-transmitted helminth (STH) infections at the 3, 6, and 12 months posttreatment follow-up time points.

### Determinants of Predisposition to Reinfection

Most studies focused on the risk of *A. lumbricoides* reinfection, particularly reinfection predisposition relating to the initial infection status, age, and sex. Seven studies were included into the pooled estimate of effect of initial infection status on *A. lumbricoides* reinfection, with a fixed RR of 1.95 (95% CI: 1.62–2.34) (*I* ^2^ = 0%, χ^2^ = 3.63, *P* = 0.73) (Figure S1) [31], [44], [48], [50], [51], [53], [54]. This means that 6 months after treatment, risk of reinfection in the pretreatment-positive group was almost twice as high than that of the pretreatment-negative group (*P*<0.001). The pooled estimates of the risk ratios of STH reinfection between subgroups are summarized in Table 2. Overall, males had a significantly lower risk of *A. lumbricoides* reinfection (*P*<0.001), and those with heavy intensity pretreatment infection with hookworm predisposed to re-acquiring high numbers of worm after therapy (*P* = 0.04) [63].

**Table 2:** Summary estimates of the risk of soil-transmitted helminth (STH) reinfection by determinants of predisposition.*

Subgroups in comparison	Risk ratios (RR) of reinfection, stratified by STH species (95% CI) (number of studies) [Reference]		
	*A. lumbricoides*	*T. trichiura*	Hookworm
Initial infection (present *vs.* absent)	1.95 (1.62–2.34) (n = 7)^F^ [31], [44], [48], [50], [51], [53], [54]	1.07 (0.41–2.80) (n = 4)^R^ [31], [51], [53], [54]	1.57 (0.74–3.37) (n = 3)^F^ [31], [51], [54]
Initial intensity (heavy *vs.* light)	3.65 (1.03–12.96, *P* = 0·05) (n = 2)^R^ [52], [63]	2.82 (0.62–12.75) (n = 1) [63]	2.55 (1.02,6.37) (n = 1) [63]
Age (adults *vs.* children)	0.76 (0.52–1.12) (n = 5)^R^ [44], [51], [54], [57], [65]	0.93 (0.31–2.81) (n = 2)^R^ [51], [54]	1.15 (0.58–2.28) (n = 2)^F^ [51], [54]
Sex (male *vs.* female)	0.71 (0.61–0.83) (n = 4)^F^ [44], [51], [54], [65]	1.06 (0.67–1.68) (n = 2)^F^ [51], [54]	1.42 (0.91–2.19) (n = 2)^F^ [51], [54]

*: Meta-analysis of nutrient supplementation, malnutrition, health promotion, individual behavior, and family and community environment was restricted because of small sample size (i.e., number of available studies for inclusion), or because of inconsistent measures used to assess infection and/or reinfection.

F Pooled estimate of fixed-effects model.

R Pooled estimate of random-effects model.

For some important outcome measures, it was not possible to perform meta-analyses because of the low number of studies addressing such outcomes and the broad differences in the measures and statistical methods used to assess them [79]. Of 16 studies reporting the predisposition to heavy or light intensity of infection after treatment, 15 provided significant statistics and one was depicted by column chart without any statistics [49]. Only two provided data of subgroups categorized by intensity level and could be pooled (Table 2) [52], [63]. Statistical tests in 15 studies consistently found a positive relationship between the intensity of infection pre- and posttreatment. Eleven were tested by Kendall's correlation [32], [35], [49], [52], [55]–[59], [62], [64], two by Pearson's [50], [53], one by Spearman [61], and one by χ^2^ [63]. Among nine studies reporting reinfection risk of infected individuals, two could not be combined due to discordant or absent statistics [49], [52]. Two studies indicated that growth-retarded children were more susceptible to STH reinfection in comparison to children with normal development [21], [39]. One randomized controlled trial indicated that multi-micronutrient fortification significantly enhanced deworming efficacy [69]. However, two randomized controlled trials separately demonstrated that supplementation of iron or multi-micronutrients in children had no significant effect, neither on prevalence nor on intensity of reinfection [19], [22], and one cross-sectional cohort study in 1- to 5-year-old children identified possible transitory benefit of vitamin A supplementation [68]. One longitudinal study in Kenyan women during pregnancy reported a positive relationship between earth-eating and STH reinfection [72]. One controlled trial in Chinese pupils assessed and quantified the impact of hand washing with soap on *A. lumbricoides* infection [71]. Four studies assessed the effect of sanitation to control STH reinfection [21], [27], [50], [73]. Three studies observed the seasonal fluctuations of *A. lumbricoides* reinfection, indicating transmission is highest when rainfall is minimal and lowest when rainfall is at its highest [29], [48].

## Discussion

Investigation of STH reinfection patterns following drug therapy dates back to the early 1920s [27]. However, nearly 90% (45/51) of studies included in the meta-analysis reported here were pursued in the past 30 years, suggesting their relevance to current deworming strategies and programs. Our broad-based meta-analysis shows that after targeted or mass drug administration, the prevalence of STH infections recovers rapidly in most endemic areas. Indeed, 6 months posttreatment, the prevalence of all three species reached or exceeded half the initial level; and at 12 months posttreatment follow-up, the prevalence of *A. lumbricoides* and *T. trichiura* usually returned to levels close to the initial pretreatment, while levels of hookworm reinfection continued to fluctuate at about half pretreatment level (Figure 4). The rate and intensity of initial pretreatment infection status were positively correlated with reinfection, although such predisposing effects were not as clear-cut for *T. trichiura* and hookworm due to limited data or discordant statistics.

### Publication and Selection Bias

We report relatively conservative pooled effect sizes, as demonstrated through sensitivity analysis. Had case studies with poor CR, low adherence with follow-up, or low coverage for treatment been included, the projected prevalence at around 6 months posttreatment would be higher than the presented pooled estimates (Table S3) [13], [28], [32], [36], [39]. One study included in our review, but not included in pooled estimates due to the difference in follow-up interval, showed that by 9 months posttreatment, re-acquired prevalence of *A. lumbricoides* and *T. trichiura* almost reached pre-intervention levels [38]. We acknowledge that some potentially relevant studies reported in Thai, Korean, Japanese, and Portuguese, identified by hand searching reference lists of included studies, were omitted. However, we are confident that excluding these studies and unpublished studies (‘grey literature’) would not impact our conservative pooled estimates (Figure 1). Our claim is substantiated by only a modest publication bias detected by funnel plots.

### Assessment of Quality of Included Studies

For our analysis, it was important to include the broadest range of data (i.e., covering a large time period and multiple locations) from field-based studies to develop a more general and inclusive assessment of the risk of STH reinfection after treatment. There was potential heterogeneity of individual study inclusion criteria, initial prevalence and intensity levels, CR, drug administration strategies, adherence, and coverage rates, as well as sanitation and risk behavior. During the process of heterogeneity testing and sensitivity analysis, it was found that a low adherence rate created outliers with a large effect on the pooled estimate. For this reason, the final exclusion threshold for adherence was set conservatively at 70%. A low initial prevalence of STH infections (<10%) also created outliers (but with little influence on estimates), while a low CR could directly limit effective measurement of reinfection rates within 6 months after drug administration. We therefore removed such cohorts from our final pooled estimates.

Most studies on reinfection of STH are based on the infected cohort (sometimes along with the uninfected cohort). Restricted by the methods of pooled analysis, the reinfection rate of such a cohort could not be combined and compared among studies. We therefore selected PRR between posttreatment and pretreatment as the indicator for estimating risk of reinfection after drug administration, which balanced the heterogeneity of studies and made them comparable. For some pooled estimates (Figure 2), we removed several outlier studies to compare the difference between fixed- and random-effects models. We found that the random estimate was similar to that of the fixed model, with wider 95% CIs than the fixed-effects model.

### Main Outcomes

In May 2001, at the 54^th^ World Health Assembly, member states were urged to pursue preventive chemotherapy to control morbidity due to STH infections and schistosomiasis, mainly among school-aged children [9], [80]–[82]. Our study indicates that in endemic areas where the prevalence of STH infections is above 10%, biannual treatment might be indicated against *A. lumbricoides* and *T. trichiura* and at least one treatment per year against hookworm. The seasonality of transmission of STHs is an important factor to consider in planning and timing of preventive chemotherapy, so that the effectiveness of this control strategy can be enhanced [13], [29], [40], [48], [83].

Our study shows that the rate and intensity of reinfection are positively correlated with the initial pretreatment infection status (Table 2). Although the dynamics of STH transmission may be intricate, reinfection levels should be expected to be similar over repeated treatments if the factors responsible for predisposition to light or heavy infection are stable through time [84]. For example, in view of PRR, there was only a modest decrease after a second-round treatment compared to the initial treatment (50.6% *vs.* 61.8% of the initial level of pretreatment for *A. lumbricoides*, 33.5% *vs.* 46.0% for *T. trichiura* [34] (for details, see Table S3). Overall, the ultimate objective of preventive chemotherapy is to control morbidity rather than to interrupt transmission of STH infections [15], [52], [81].

### Limitations

There are limitations to this systematic review and meta-analysis, which are offered for consideration. First, epidemiological and statistical heterogeneity between studies allows possible confounding of the observed results. For example, differences in age, sex, socioeconomic and nutritional status could modify the risk of STH reinfection, which could then confound estimation of the effects of these determinants. However, details of these potentially modifying factors were not available in most of the studies included in our analysis, so adjustment was not attempted in the summary statistics. Other factors, such as diagnostic method, the frequency, approach, and efficacy of anthelmintic drugs, and length and adherence of follow-up, also varied between studies. Second, for continuous distributions, relatively large changes in average worm load could have been related to only small changes in overall prevalence such that prevalence, and hence CR, may not be the best indicator to monitor the impact of anthelmintic treatment in highly endemic areas [33], [40], [52], [76]. Indeed, it has been argued that egg reduction rate (ERR) rather than CR should be used for anthelmintic drug efficacy evaluations [85]–[87], and perhaps for studying patterns of reinfection after treatment. Intensity of infection is an important aspect of all helminthiases, but could not be well addressed in our pooled estimates. Similarly, the significant relationships between STH reinfection and socioeconomic factors could not be definitively assessed [4]. Finally, the generalized estimates from our study will probably not apply to low endemicity areas, characterized by prevalence estimates below 10%.

Concluding, preventive chemotherapy with the current drugs of choice against STHs, while showing good results on morbidity reduction, does not prevent rapid reinfection [88]. Decision-makers have to make tradeoffs between benefits, cost (selected *vs.* mass treatment), acceptability, and harms (e.g., drug resistance) of preventive chemotherapy according the local settings [89]–[92]. Our results therefore support recommendations for integrated control approaches, complementing preventive chemotherapy with information, education, and communication (IEC) strategies, and sanitation improvement, in order to control STH infections more durably [6], [93]–[95]. The experience from the southern parts of the United States of America almost 100 years ago, as well as the Republic of Korea, P.R. China, and some parts of sub-Saharan Africa indicates that control strategies must be adapted to the prevailing social-ecological setting, and require long-term political commitment [95], [96]. In resource-limited settings, regular deworming of school-aged children is considered to be a cost-effective intervention for control of morbidity due to STH infections [9], [80], [96]. More intense treatment (e.g., twice yearly) is likely to further impact on morbidity, as seen for schistosomiasis [90], but might bear the risk of drug resistance development [91], [97]. If resources allow, efforts should be made to promote clean water, improved sanitation, health promotion in schools, and these efforts should ultimately aim at behavioral change [6], [98]–[101]. Communities living in newly industrialized countries such as P.R. China, should benefit from an integrated treatment and sanitation strategy. Hence, rather than solely being restricted to preventive chemotherapy targeting STHs and other neglected tropical diseases [102], comprehensive control requires inter-programmatic and intersectoral action for health and development [103], [104].

## Supporting Information

Figure S1 **Forest plot of reinfection risk of individuals initially infected with** ***Ascaris lumbricoides*** **, 6–12 months posttreatment.** Notes: A random relative risk (RR) of less than 1 indicates a lower infection rate after treatment compared to the initial level. Diamonds represent the pooled estimate across studies. See Table S1 for full references.
(PDF)

Table S1 **Included studies.**
(DOC)

Table S2 **Excluded studies.**
(DOC)

Table S3 Studies included in our meta-analysis pertaining to reinfection patterns of soil-transmitted helminths (STHs) 3–12 months posttreatment.^*^
(DOC)

Checklist S1 **PRISMA checklist.**
(DOC)
