# Supplementary figures and images for: Soil-Transmitted Helminth Reinfection after Drug Treatment: A Systematic Review and Meta-Analysis
Source: PLoS Negl Trop Dis. 2012 May 8;6(5):e1621. doi: 10.1371/journal.pntd.0001621 (PMC3348161; doi:10.1371/journal.pntd.0001621)

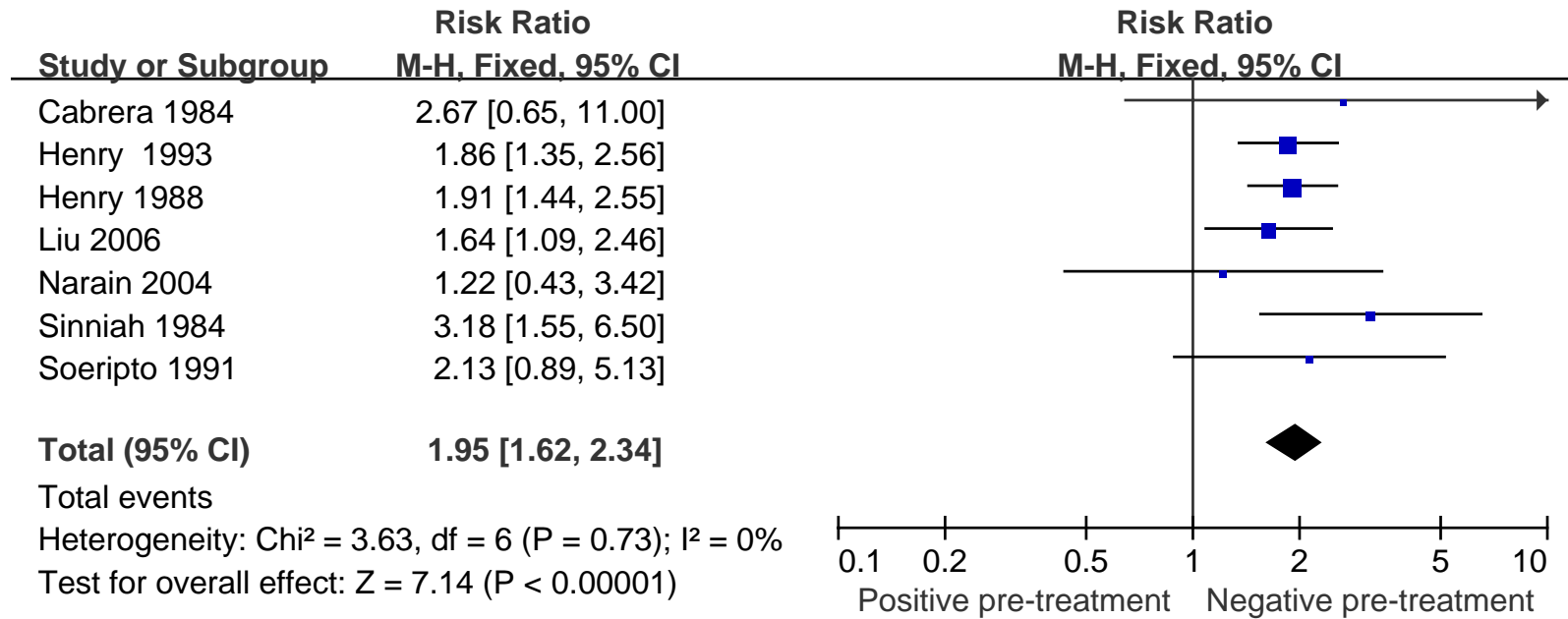

Supplement: Figure S1 — Forest plot of reinfection risk of individuals initially infected with Ascaris lumbricoides , 6–12 months posttreatment. Notes: A random relative risk (RR) of less than 1 indicates a lower infection rate after treatment compared to the initial level. Diamonds represent the pooled estimate across studies. See Table S1 for full references. (PDF) [file pntd.0001621.s001.pdf]
