# Supplementary material for: Soil-Transmitted Helminth Reinfection after Drug Treatment: A Systematic Review and Meta-Analysis
Source: PLoS Negl Trop Dis. 2012 May 8;6(5):e1621. doi: 10.1371/journal.pntd.0001621 (PMC3348161; doi:10.1371/journal.pntd.0001621)
Supplement: Table S1 — Included studies. (DOC) [file pntd.0001621.s002.doc]

**Table S1**: Included studies

| **First Author, year** | **Citation** | **Location of trial** | **Species of STH *** |
| --- | --- | --- | --- |
| Appleton CC, 2009 | Ann Trop Med Parasitol 103(3): 249-61 | South Africa | A, T, H |
| Nchito M, 2009 | Trans R Soc Trop Med Hyg 103(3): 229-36 | Zambia | A |
| Nga TT, 2009 | J Nutr 139(5): 1013-1021 | Vietnam | A, T, H |
| Hesham Al-Mekhlafi M, 2008 | Trans R Soc Trop Med Hyg 103(3): 229-36 | Malaysia | A, T, H |
| Payne LG, 2007 | J Nutr 137(6): 1455-9 | Panama | A |
| Zhang Y, 2007 | BMC Med 5: 27 | Uganda | A, T, H |
| Liu CY, 2006 | J of Pathogen Biology 1(6): 449-451 | China | A |
| Fei YL, 2006 | Chin J School Doctor 20(5): 522, 523 | China | A |
| Louba AI, 2005 | Tropical Medicine and International Health 10(3): 220-227 | Kenya | A, T, H |
| Saathoff E, 2004 | BMC Infect Dis 4: 27 | South Africa | A, T, H |
| Narain K, 2004 | Southeast Asian J Trop Med Public Health 35(3): 512-7 | India | A, T, H |
| Olsen A, 2003 | Trans R Soc Trop Med Hyg 97(1): 109-14 | Kenya | A, T, H |
| Han GT, 2003 | Chin J School Health 24(4): 358 | China | A, H |
| Peng YK 2001 | Chin J of Schisto Contr 13(3): 156 | China | A |
| Xu LQ, 2001 | Chin J Parasit Dis Con 19(5): 294-7 | China | A |
| Olsen A, 2000 | Trans R Soc Trop Med Hyg 94(5): 493-9 | Kenya | A, T, H |
| Feng XP, 2000 | J Pre Med Chin PLA 18(3): 210-211 | China | A, T, H |
| Hagel I, 1999 | J Trop Pediatr 45(4): 215-20 | Venezuela | A, T |
| Paul I, 1999 | Indian J Pediatr 66(5): 669-73 | India | A, T, H |
| Albonico M, 1999 | Int J Epidemiol 28(3): 591-6 | Tanzania | A, T, H |
| Muennoo C, 1997 | Southeast Asian J Trop Med Public Health 28(4): 816-9 | Thailand | A, T, H |
| Albonico M, 1995 | Trans R Soc Trop Med Hyg 89(5): 538-41 | Tanzania | A, T, H |
| Henry FJ, 1993 | Southeast Asian J Trop Med Public Health 24(2): 307-12 | Bangladesh | A, T, H |
| Quinnell RJ, 1993 | Parasitology 106 ( Pt 4): 379-85 | PN Guinea | H |
| Upatham ES, 1992 | Int J Parasitol 22(6): 801-6 | Thailand | A, T, H |
| Hall A, 1992 | Lancet 339(8804): 1253-7 | Bangladesh | A |
| Chan L, 1992 | Southeast Asian J Trop Med Public Health 23(2): 228-34 | Malaysia | A, T |
| Soeripto N, 1991 | Southeast Asian J Trop Med Public Health 22(2): 216-21 | Indonesia | A, T, H |
| Forrester JE, 1990 | Trans R Soc Trop Med Hyg 84(2): 272-6 | Mexico | A, T |
| Bradley M, 1990 | Trans R Soc Trop Med Hyg 84(6): 826-8 | Zimbabwe | H |
| Haswell-Elkins M, 1989 | Parasitology 98 ( Pt 1): 125-34 | India | A |
| Henry FJ, 1988 | Trans R Soc Trop Med Hyg 82(3): 460-4 | St Lucia | A |
| Haswell-Elkins MR, 1988 | Parasitology 96 ( Pt 3): 565-77 | India | H |
| Elkins DB, 1988 | Parasitology 96 ( Pt 1): 171-84 | India | A |
| Bundy DA, 1988 | Parasitology 97 ( Pt 3): 469-76 | St Lucia | T |
| Bundy DA, 1988 | Ann Trop Med Parasitol 82(3): 251-6 | St Lucia and Jamaica | T |
| Hlaing T, 1987 | Trans R Soc Trop Med Hyg 81(1): 140-6 | Myanmar | A |
| Haswell-Elkins MR, 1987 | Parasitology 95 ( Pt 2): 323-37 | India | A, T, H |
| Bundy DA, 1987 | Trans R Soc Trop Med Hyg 81(6): 987-93 | St Lucia | A, T |
| Bundy DA, 1987 | Epidemiol Infect 98(1): 65-71 | St Lucia | T |
| Schad GA, 1985 | Science 228(4707): 1537-40 | India | H |
| Imai J, 1985 | Southeast Asian J Trop Med Public Health 16(3): 441-6 | Indonesia | A, T, H |
| Cabrera BD 1984 | Southeast Asian J Trop Med Public Health 15(3): 394-401 | Philippines | A |
| Sinniah B 1984 | Public Health 98(1): 38-42 | Malaysia | A, T, H |
| Chai JY, 1983 | Korean J Parasitol 21(2): 142-149 | Korea | A |
| Arfaa F, 1977 | Am J Trop Med Hyg 26(5 Pt 1): 866-71 | Iran | A |
| Pan CT, 1954 | J Parasitol 40(5, Part 1): 603-8 | Japan | A |
| Otto GF, 1934 | J Parasitol 20(4): 245-247 | USA | A |
| Otto GF, 1930 | JAMA 95(3): 194-196 | USA | A |
| Cort WW, 1929 | Am J Epidemiol 10: 614-625 | Panama | A, H |
| Hill RB, 1925 | Southern Med J 18(9): 665-668 | Porto Rico | H |

^*^Species of soil-transmitted helminths: A, *Ascaris lumbricoides*; T, *Trichuris trichiura*; H, hookworm.
