# Supplementary material for: Soil-Transmitted Helminth Reinfection after Drug Treatment: A Systematic Review and Meta-Analysis
Source: PLoS Negl Trop Dis. 2012 May 8;6(5):e1621. doi: 10.1371/journal.pntd.0001621 (PMC3348161; doi:10.1371/journal.pntd.0001621)
Supplement: Table S2 — Excluded studies. (DOC) [file pntd.0001621.s003.doc]

**Table S2**: Excluded studies

| **First Author, year** | **Citation** | **Helminth species***^＊^* |
| --- | --- | --- |
| Casey GJ, 2010 | PLoS ONE 5(12): e15691 | A, T, H |
| Knopp S, 2010 | Clin Infect Dis 51(12): 1420-1428 | T |
| Kurup R, 2010 | J Rural and Tropical Public Health 9: 24-30 | A, T, H |
| Kurup R, 2010 | J Vector Borne Dis 47(4): 228-234 | A, T, H, S |
| Nkengazong L, 2010 | Afr J Environ Sci Technol 4(3): 115-121 | A, T, H |
| Ohnmacht C, 2010 | J Immunol 184(1): 344-50 | H |
| Quinnell RJ, 2010 | J Infect Dis 202(6): 954-61 | H |
| Shaw JG, 2010 | Am J Trop Med Hyg 83(2): 416-21 | A, T, H |
| Uneke CJ 2010 | Tanzan J Health Res 12(1): 86-99 | A, T, H |
| Walker M, 2010 | Int J Parasitol 40(12): 1373-80 | A |
| Massa K, 2009 | Trans R Soc Trop Med Hyg 103(1): 31-7 | A, T, H |
| Mihrshahi S, 2009 | Int J Parasitol 39(9): 1037-1043 | A, T, H |
| Kirwan P, 2009 | BMC Infect Dis 9: 20 | A, T, H, S |
| Knopp S, 2009 | Am J Trop Med Hyg 81(6): 1071-8 | A, T, H |
| Pogonka T, 2009 | Microbes Infect 12(3): 218-26 | Ef |
| Toledo MJ, 2009 | Rev Saude Publica 43(6): 981-90 | A, T, Ent |
| Moncayo AL, 2008 | PLoS Negl Trop Dis 2(9): e293 | A, T, H, Onc. |
| Gunawardena NK, 2008 | Ceylon Med J 53(1): 13-6 | A, T, H |
| Mukhopadhyay C, 2008 | BMC Public Health 8: 98 | A, T, H |
| Yang WP, 2008 | J Pathogen Biology 3(7): FuYe3, 01 | H |
| Gungoren B, 2007 | Trans R Soc Trop Med Hyg 101(6): 564-9 | Ent |
| Kabatereine NB, 2007 | Bull World Health Organ 85(2): 91-9 | A, T, H, S |
| Kihara JH, 2007 | Acta Trop 102(3): 165-71 | A, T, H |
| Sam-Wobo SO, 2007 | Rev Biol Trop 55(3-4): 755-60 |  |
| Boia MN, 2006 | Rev Inst Med Trop Sao Paulo 48(4): 189-95 | A, T, H |
| Ramzy RM, 2006 | Lancet 367(9515): 992-9 | F |
| Poggensee G, 2005 | Acta Trop 93(2): 131-40 | A, T, H, S |
| Saathoff E, 2005 | Am J Trop Med Hyg 72(4): 384-91 | H |
| Oqueka T, 2005 | Filaria J 4: 5 | A, T, H, F |
| Chen YD, 2005 | Chin J Parasit Dis Con 18(2): 132-133 | A |
| Yang WP, 2004 | Chin J Parasit Dis Con 17(5): 279-281 | A, T, H, E |
| Morrone FB, 2004 | Rev Inst Med Trop Sao Paulo 46(2): 77-80 | A, T |
| Quinnell RJ, 2004 | J Infect Dis 190(3): 430-8 | Nec |
| Uga S, 2004 | Jpn J Environ Entomol Zool 15(4): 225-229 | A, T, H, E |
| Belizario VY, 2004 | Southeast Asian J Trop Med Public Health 35(Sup1): 123-39 | STH, Het |
| Albonico M, 2003 | Bull World Health Organ 81(5): 343-52 | A, T, H |
| Tu XG, 2003 | Chin J Parasitol Parasit Dis 21(5): 307-310 | A, T, H, E |
| Peng W, 2002 | Parasitology 124(Pt 6): 641-7 | A |
| Saldiva SR, 2002 | Paediatr Perinat Epidemiol 16(2): 166-71 | Ent |
| Rajendran R, 2003 | Trans R Soc Trop Med Hyg 97(3): 355-9 | A, T, H |
| Guyatt HL, 2001 | Bull World Health Organ 79(8): 695-703 | H, S |
| Muchiri EM, 2001 | J Parasitol 87(2): 413-418 | A, T, H |
| Quinnell RJ, 2001 | Trans R Soc Trop Med Hyg 95(2): 139-42 | H |
| Taylor M, 2001 | Trans R Soc Trop Med Hyg 95(2): 211-6 | A, T, H |
| Zeng XJ, 2001 | Chin J of Parasitic Disease Control 14(4): 265-267 | A, T, H |
| Peng W, 1998 | J Parasitol 84(2): 252-8 | A |
| Paul I, 1998 | J Commun Dis 30(4): 245-9a | A, T, H |
| Norhayati M, 1997 | Southeast Asian J Trop Med Public Health 28(3): 563-9 | A, T, H |
| Sun FH, 1994 | Chin J Parasit Dis Con 19(5): 294-7 | A |
| Guyatt HL, 1993 | Trans R Soc Trop Med Hyg 87(5): 570-5 | A |
| Hagel I, 1993 | Clin Exp Immunol 94(1): 80-3 | A, T, H |
| Jiang H, 1992 | Chin J of Parasitology and Parasitic Diseases 10(1): 69 | H |
| Chan L, 1992 | Parasitology 104 ( Pt 2): 371-7 | A, T |
| Pritchard DI, 1992 | Parasite Immunol 14(6): 617-31 | H |
| Thein H, 1991 | Trans R Soc Trop Med Hyg 85(4): 519-22 | A |
| Whitworth JA, 1991 | Trans R Soc Trop Med Hyg 85(2): 232-4 | A, T, H, S |
| Thein H, 1990 | Bull World Health Organ 68(6): 747-53 | A |
| Shiddo S, 1990 | Trans R Soc Trop Med Hyg 84(6): 832-6 | H |
| Haswell-Elkins M, 1989 | Parasitology 98 ( Pt 1): 125-34 | A |
| Upatham ES, 1989 | Int J Parasitol 19(2): 217-28 | A, T, H |
| Elkins DB, 1989 | Parasitology 99 Pt 2: 293-9 | A |
| Bundy DA, 1988 | Philos Trans R Soc Lond B Biol Sci 321(1207): 405-20 |  |
| Bundy DA, 1987 | Trans R Soc Trop Med Hyg 81(1): 85-94 | T |
| Behnke JM, 1987 | Parasit Today 3(7): 200-206 | H |
| Elkins DB, 1986 | Trans R Soc Trop Med Hyg 80(5): 774-92 | A |
| Anderson RM, 1986 | Trans R Soc Trop Med Hyg 80(5): 686-96 | Hel |
| Bundy DA, 1986 | Trans R Soc Trop Med Hyg 80(5): 706-18 | T |
| Anderson RM, 1985 | Nature 315(6019): 493-6 | Hel |
| Bundy DA, 1985 | Trans R Soc Trop Med Hyg 79(6): 759-64 | H |
| Anderson RM, 1985 | Parasitology 90 ( Pt 4): 629-60 |  |
| Anderson RM, 1985 | Adv Parasitol 24: 1-101 | Hel |
| Thein H, 1984 | Trans R Soc Trop Med Hyg 78(4): 497-504 | A |
| Shield J, 1984 | P N G Med J 27(2): 89-94 | A, T, H |
| Chai JY, 1983 | Korean J Parasitol 21(2): 135-141 | A |
| Seo BS, 1983 | APCO Research Group. 11: 194-216 | A |
| Yodmani B, 1983 | Collected Papers on the Control of Soil-transmitted Helminthiases [APCO]. Tokyo, The Asian Parasite Control Organization. 2: 89-100. | A |
| Anderson RM, 1982 | Nature 297(5867): 557-63 | Hel |
| Croll NA, 1982 | Trans R Soc Trop Med Hyg 76(2): 187-197 | A |
| Henry FJ 1981 | Trans R Soc Trop Med Hyg 75(4): 507-13 | A, T |
| Seo BS, 1980 | Korean J Parasitol 18(2): 145-151 | A |
| Seo BS, 1980 | Korean J Parasitol 18(2): 153-163 | A |
| Cabrera BD, 1980 | Collected papers on the control of soil-transmitted helminthiases. Tokyo, Asian Parasite Control Organization [APCO]. 1: 181-191 |  |
| Cabrera BD, 1980 | Collected papers on the control of soil-transmitted helminthiases. Tokyo, Asian Parasite Control Organization [APCO]. 1: 141-9 | A, T, H |
| Rose ME, 1979 | Parasite Immunol 1(2): 125-32 | En or Nb |
| Cabrera BD, 1978 | Acta Med Philipp 14: 8 |  |
| Cabrera BD, 1978 | The Fourth Conference of the Asian Parasite Control Organization--the Integrated Family Planning Project--Tokyo, October 3-8, 1977: proceedings. Tokyo, APCO**:** 237-54. |  |
| Cho SY 1977 | Korean J Parasitol 15(1): 17-29 | A |
| Cabrera BD, 1975 | Southeast Asian J Trop Med Public Health 6(4): 510-8 | A |
| Sargent RG, 1972 | South Med J 65(3): 294-8 | A, T, H |
| Morishita K, 1972 | Progress of Medical Parasitology in Japan 5: 145-213 |  |
| Jueco NL, 1971 | J Phil. Med. Ass. 47: 449 | A |
| Lease EJ, 1970 | J S C Med Assoc 66(10): 359-62 | A |
| Garcia EG, 1961 | J Phil. Med. Ass. 37: 239 | A |
| Pan C, 1953 | Med Bull US Army, Far East 1: 49-51 | A |
| Caldwell FC, 1931 | J Parasitol 17(4): 209-222 | H |
| Schapiro L, 1930 | Am J Epidemiol 12: 699-708 | A, T, H |
| Cort WW, 1929 | Am J Epidemiol 10(3): 626-634 | A, T, H |
| Hill RB, 1926 | Am J Epidemiol 6(supp2): 103-117 | H |
| Docherty JF, 1926 | Am J Epidemiol 6(suppl): 160-171 | H |
| Chandler AC, 1926 | Ind J Med Res 13: 625-634 | H |
| Sweet WC, 1925 | Ceylon J Sci D 1: 129-140 | H |
| Cort WW, 1923 | Am J Epidemiol 3(supp2): 85-110 | H |
| Hill RB, 1923 | Am J Epidemiol 3(supp2): 37-60 | H |

^*^Species of helminths: A, *Ascaris lumbricoides*; T, *Trichuris trichiura*; H, hookworm; S, *Schistosoma*; STH, soil-transmitted helminths, F, filaria; E, *Enterobius vermicularis*.

Other: Onc, Onchocerciasis; Ent, other enteroparasites; Ef, Eimeria falciformis; Nec, Necatoriasis; Het, Heterophyid infections; Hel, helminths in general; En, Eimeria nieschulzi; Nb, the nematode Nippostrongylus brasiliensis.
