# Supplementary material for: Soil-Transmitted Helminth Reinfection after Drug Treatment: A Systematic Review and Meta-Analysis
Source: PLoS Negl Trop Dis. 2012 May 8;6(5):e1621. doi: 10.1371/journal.pntd.0001621 (PMC3348161; doi:10.1371/journal.pntd.0001621)
Supplement: Table S3 — Studies included in our meta-analysis pertaining to reinfection patterns of soil-transmitted helminths (STHs) 3–12 months posttreatment.* (DOC) [file pntd.0001621.s004.doc]

**Table S3. Studies included in our meta-analysis pertaining to reinfection patterns of soil-transmitted helminths (STHs) 3-12 months posttreatment^*^**

| **Source (location, year of trial)** | **Age of participants (year)** | **Sample size** | **Diagnostic approach (number of samples)** | **SDA (U/T/S)** | **Follow–up interval**** | **STH** |  | **Before treatment** | | **Cure rate (%) ***[Ref.]** |  | **After treatment** | | | | | |
| --- | --- | --- | --- | --- | --- | --- | --- | --- | --- | --- | --- | --- | --- | --- | --- | --- | --- |
|  |  |  |  |  |  |  |  | **P (%)** | **II (EPG)** |  |  | **Time** | **Prevalence** | |  | **Intensity** | |
|  |  |  |  |  |  |  |  |  |  |  |  |  | **P (%)** | **PRR (%)** |  | **II (EPG)** | **IIR (%)** |
| Appleton, 2009 (South Africa, 1998–2000) [47] | 2–10 | 996 | Kato–Katz technique (2) | ALB 400 mg, single dose (T) | 1 m 4–6 m, and 12 m | *A. l.* |  | 89.2 | 8812^GM1^ | 95.6^a^ |  | 4–6 m | 64.0 | 71.7 |  | 188 | 2.1 |
|  |  |  |  |  |  |  |  |  |  |  |  | 12 m | 87.5 | 98.1 |  | 4834 | 54.9 |
|  |  |  |  |  |  | *T. t.* |  | 71.6 | 128^GM1^ | 67.6^a^ |  | 4–6 m | 43.6 | 60.9 |  | 12 | 9.3 |
|  |  |  |  |  |  |  |  |  |  |  |  | 12 m | 70.7 | 98.7 |  | 212 | 165.6 |
| Nchito, 2009 (Zambia, 2001) [46] | 7–15 | 378 | Kato–Katz technique (2) | ALB 400 mg/d*2 d (S) | 3 w; 6 m, and 10 m | *A. l.* |  | 43.4 | 2526^GM2^ | 89^a^ |  | 6 m | 31.0 | 71.4 |  | NA |  |
|  |  |  |  |  |  |  |  |  |  |  |  | 10 m | 45.0 | 103.7 |  |  |  |
| Zhang, 2007 (Uganda, 2003–2005) [45] | 6–14 | 1704 | Kato–Katz technique (all slides read within 2 h of preparation) | ALB 400 mg single dose, annually (T) | 12 m and 2 y | Hw |  | 50.9 | 309.4^AM1^ | 72 [18] |  | 1 y | 24.1 | 47.3 |  | 76.8 | 24.8 |
|  |  |  |  |  |  |  |  |  |  |  |  | 2 y | 10.7 | 21.0 |  | 21.9 | 7.1 |
| Liu, 2006 (P.R. China, 1999–2000) [44] | 5–65 | 134 | Kato–Katz technique (1) | PYR 10 mg/kg single dose, at 3, 6, and 12 m interval for a year (U) | 12 m | *A. l.*^4R^ |  | 54.5 | 29.4^GM1^ | NA^a^ |  | 3 m | 25.4 | 46.6 |  | 4.03 | 13.7 |
|  |  | 69 |  |  |  | *A. l.*^2R^ |  | 44.9 | 11.5^GM1^ | NA^a^ |  | 6 m | 26.1 | 58.1 |  | 4.67 | 40.6 |
|  |  | 156 |  |  |  | *A. l.*^1R^ |  | 53.9 | 29.7^GM1^ | NA^a^ |  | 12 m | 41.7 | 77.4 |  | 15.13 | 51.0 |
| Fei, 2006 (P.R. China, 2004) [43] | School–aged children | 317 | Saturated saline flotation technique | LEV 75–150 mg single dose (T) | 2 m, 3 m, and 6 m | *A. l.* |  | 72.6 | NA | 95.0 |  | 3 m | 10.8 | 14.9 |  | NA |  |
|  |  |  |  |  |  |  |  |  |  |  |  | 6 m | 39.7 | 54.7 |  |  |  |
| Saathoff, 2004 (South Africa, 1998) [42] | 9–13 | 1017 | Kato–Katz technique (1 at baseline, 2 at follow-up examinations); hookworm eggs examined within 1 h of preparation) | ALB 400 mg singledose, twice a year (T) | 18 and 29 w after 2nd treatment | *A. l.* |  | 19.4 | 2041^AM1^ | 97.7^2T^ |  | 4 m | 3.0 | 15.5 |  | 59 | 2.9 |
|  |  |  |  |  |  |  |  |  |  |  |  | 7 m | 8.6 | 44.3 |  | 351 | 17.2 |
|  |  |  |  |  |  | *T. t.* |  | 57.2 | 292^AM1^ | 33.3^2T^ |  | 4 m | 44.8 | 78.3 |  | 174 | 59.6 |
|  |  |  |  |  |  |  |  |  |  |  |  | 7 m | 43.4 | 75.9 |  | 175 | 59.9 |
|  |  |  |  |  |  | Hw |  | 83.2 | 876^AM1^ | 93.2^2T^ |  | 4 m | 24.6 | 29.6 |  | 65 | 7.4 |
|  |  |  |  |  |  |  |  |  |  |  |  | 7 m | 36.7 | 44.1 |  | 139 | 15.9 |
| Han, 2003 (P.R. China, NA) [41] | School–aged children | 501 | Kato–Katz technique, supplemented with culture method for hookworm larvae detection | ALB 400 mg single dose (T) | 1 m, 6 m | *A. l.* |  | 39.7 | NA | 92.0 |  | 6 m | 29.3 | 73.8 |  | NA |  |
|  |  |  |  |  |  | Hw |  | 19.4 |  | 95.4 |  | 6 m | 13.7 | 70.6 |  |  |  |
| Olsen, 2003 (Kenya, 1995-1996) [22] | 8–18 | 977 | Kato-Katz technique (2-3; hookworm eggs examined within 1 h of preparation) | ALB 600 mg single dose (S) | 4 w, 11 m | *A. l.* |  | 13.8 | 2.7^GM1^ | 96.9^a^ |  | 11 m | 13.3 | 96.4 |  | 2.3 | 85.1 |
|  |  |  |  |  |  | *T. t.*^LA^ |  | 45.6 | 5.9^GM1^ | 44.0^a^ |  | 11 m | 19.2 | 42.1 |  | 1.9 | 32.2 |
|  |  |  |  |  |  | Hw^LA^ |  | 54.7 | 8.6^GM1^ | 90.9^a^ |  | 11 m | 18.7 | 34.2 |  | 6.5 | 75.6 |
| Olsen, 2000 (Kenya, 1994–1996) [19] | 4–15 | 200 | Kato–Katz technique (2; hookworm eggs examined within 1 h of preparation) | ALB 400 mg/d * 3 d (S) | 3–6 w; 12 m | *A. l.* |  | 27.0 | NA | 100^a^ |  | 12 m | 40.5 | 150 |  | NA |  |
|  |  |  |  |  |  | *T. t.* |  | 39.5 |  | 69.0^a^ |  | 12 m | 29.6 | 75 |  |  |  |
|  |  |  |  |  |  | Hw |  | 60.0 |  | 94.0^a^ |  | 12 m | 36.0 | 60 |  |  |  |
| Albonico, 1999 (Tanzania, 1994–1995) [40] | 10·6±2·0 | 922 | Kato–Katz technique plus modified Stoll dilution for egg counts >10,000 EPG | MEB 500 mg, single dose, twice a year (T) | 6, 12 m | *A. l.* |  | 66.5 | 149^GM1^ | 95 [18] |  | 6 m | 58.5 | 88.0 |  | 55 | 36.9 |
|  |  |  |  |  |  | *T. t.* |  | 96.6 | 584^GM1^ | 36 [18] |  | 6 m | 92.0 | 95.2 |  | 348 | 59.6 |
| Hagel, 1999 (Venezuela, NA)^LA^ [39] | 8.5±2.5 | 154 | Merthiolate–iodine–formaldehyde sedimentation technique (≥3) | Oxantel/pyrantel 20 mg/kg monthly for 12 m (T) | 8 m | *A. l.* |  | 59.1 | NA | NA [18] |  | 8 m | 55.3 | 93.6 |  | NA |  |
| Paul, 1999 (India, 1993–1994) [38] | 7–13 | 217 | Formalin–ethyl–acetate sedimentation technique (1) | ALB 400 mg, single dose (T) | 1 m, 9 m | *A. l.* |  | 73 | 3413^NA^ | 100 |  | 9 m | 72 | 98.6 |  | NA |  |
|  |  |  |  |  |  | *T. t.* |  | 66 | 918^NA^ | 28 [18] |  | 9 m | 68 | 103.0 |  |  |  |
|  |  |  |  |  |  | Hw |  | 9 | 165^NA^ | 100 |  | 9 m | 1 | 11.1 |  |  |  |
| Muennoo, 1997 (Thailand, NA) [37] | All ages | 389 | Kato–Katz technique | ALB 400 mg single dose (S) (400 mg/d * 3 d for *T. t.*) | 21 d; 12 m | *A. l.* |  | 17.5 | NA | NA^a^ |  | 12 m | 6.7 | 38.3 |  | NA |  |
|  |  |  |  |  |  | *T. t.* |  | 60.4 |  |  |  | 12 m | 43.0 | 71.2 |  |  |  |
|  |  |  |  |  |  | Hw |  | 68.6 |  |  |  | 12 m | 38.2 | 55.7 |  |  |  |
| Albonico, 1995 (Tanzania, 1992-1993)^LA^ [36] | 6–12 | 731 | Kato-Katz technique, and modified Stoll dilution egg counting for egg count > 10,000 eggs/g (1); hookworm eggs examined within 1 h of preparation [74] | ALB 400mg or MEB 500 mg single dose | 3 w; 4 and 6 m | *A. l* |  | 67 | NA | 98.9^ALB^; 97.8^MEB^ [74] |  | 6 m | 72 | 107.5 |  | NA | 81.3^ALB^;  75.9^MEB[GM1]^ |
|  |  |  |  |  |  | *T. t.* |  | 97 |  | 10.5^ALB^; 14.2^MEB^ [74] |  | 6 m | 97 | 100.0 |  |  | 130.5^ALB^;  94.3^MEB[GM1]^ |
|  |  |  |  |  |  | Hw |  | 92 |  | 56.8^ALB^; 22.4^MEB^ [74] |  | 6 m | 95 | 103.3 |  |  | 154.5^ALB^; 117.9^MEB[GM1]^ |
| Quinnell, 1993 (Papua New Guinea, 1988–1990) [35] | All ages | 140 | Modified formalin–ether sedimentation technique at baseline (1) [75] | PYR 10 mg/kg single dose (U) | 1, 2y | Hw |  | 92.9 | 93^GM1^ | 60.0 |  | 1 y | 65.0 | 70.0 |  | 23 | 24.7 |
|  |  |  |  |  |  |  |  |  |  |  |  | 2 y | 80.7 | 86.9 |  | 45 | 48.4 |
| Chan, 1992 (Malaysia, 1989–1990) [34] | All ages | 589 | Kato–Katz technique (1) | ALB 400 mg/d * 3 d, 3 rounds at 6 m interval (U) | 7 d; 6 m after each round | *A. l.* |  | 30.6 | NA | 100 |  | 6 m^1R^ | 18.9 | 61.8 |  | NA |  |
|  |  |  |  |  |  |  |  |  |  |  |  | 6 m^2R^ | 15.5 | 50.6 |  |  |  |
|  |  |  |  |  |  | *T. t.* |  | 46.9 |  | 67.5 |  | 6 m^1R^ | 21.6 | 46.0 |  |  |  |
|  |  |  |  |  |  |  |  |  |  |  |  | 6 m^2R^ | 15.7 | 33.5 |  |  |  |
| Elkins, 1988 (India, 1984) [33] | All ages | 325 | Modified formalin–ethyl acetate sedimentation technique (1) [76] | PYR 10 mg/kg single dose (U) | 2 m; 6,11 m | *A. l.* |  | 85 | NA | 95 [76] |  | 11 m | 75 | 88.2 |  | NA |  |
| Haswell-Elkins, 1988 (India, 1984)^LA^ [32] |  |  |  |  |  | Hw ^LA^ |  | 31 | 73^NA^ | 91 |  | 6 m | 20.6 | 66.4 |  | NA (expressed by fig.) | Approximately 50 (extracted from Figure) |
| Sinniah, 1984 (Malaysia, NA) [31] | 6–13 | 389 | Brine–flotation method and Beaver's egg count (1) | PYR 10 mg/kg single dose (S) | 3 w; 15 w | *A. l.* |  | 37·5 | 23,612^AM2^ | 89.7 |  | 15 w | 17.7 | 47.2 |  | NA |  |
|  |  |  |  |  |  | *T. t.* |  | 53·7 | 2926^AM2^ | 56.4 |  | 15 w | 19.4 | 36.1 |  |  |  |
|  |  |  |  |  |  | Hw |  | 5·4 | 1876^AM2^ | 44.4 |  | 15 w | 6.9 | 127.8 |  |  |  |
| Arfaa, 1977 (Iran, 1972–1973) [30] | All ages | 1455 | Formalin–ether concentration technique and Stoll dilution egg counting | PYR 10 mg/kg, single dose (S) | 2–3 w; 2, 4, 6 and 12 m | *A. l.* |  | 91 | 20,000^NA^ | 95 |  | 1 y | 87 | 95.6 |  | 10,000 | 50.0 |
| Pan, 1954 (Japan, 1950–1951) [29] | 12–15 | 282 | AMS III centrifugation-sedimentation technique (1) [77] | Hexylresorcinol 1 g single dose (S) | 2–4 w; 2–14 m, monthly | *A. l.* |  | 78 | NA | 100^b^ |  | 3 m | 17 | 21.8 |  | NA |  |
|  |  |  |  |  |  |  |  |  |  |  |  | 6 m | 59 | 75.6 |  |  |  |
| Otto, 1934 (United States of America, 1930-1931)^PC^ [28] | School-aged children | 102 | Stoll dilution egg counting | Hexylresorcinol at various doses | 2-3 w; 5 and 8 m | *A. l.* |  | 100 | 23,000^AM1^ | 48 |  | 5 m | 78 | 78.0 |  | 17,100 | 74.3 |
|  |  |  |  |  |  |  |  |  |  |  |  | 8 m | 85 | 85.0 |  | 33,000 | 143.5 |
| Otto, 1930 (United States of America, 1928-1929)^PC^ [13] | All ages | 157 | Stoll dilution egg counting | Chenopodium | 1 m; 11 m | *A. l.* |  | 69.4 | 22,000^AM1^ | 35.7 |  | 11 m | 80.0 | 115.3 |  | 24,400 | 110.9 |
| Hill, 1925 (Porto Rico, 1922–1923) [27] | All ages | 282 | Smear, centrifugation, and Stoll dilution egg counting [78] | Chenopodium and thymol, weekly (S) | 7 d and more; 1 y | Hw |  | 89.7 | 2820^AM1^ | 82.1 |  | 1 y | 51.5 | 57.4 |  | 338 | 12.0 |

**Abbreviations:**

NA, not available; MDA, mass drug administration; SDA, strategy of drug administration: 1) universal treatment (U), sometimes referred to as mass or blanket treatment, indicates population level application of the drug in which everybody is treated irrespective of age, sex, occupation, infection status, or social characteristics; 2) targeted treatment (T) indicates group-level application of anthelmintic drugs where the group eligible for treatment may be defined by age, sex, or other social characteristics irrespective of infection status; 3) selective treatment (S) indicates individual application of the drug after determination of infection status. ALB, albendazole; MEB, mebendazole; PYR, pyrantel pamoate; LEV, levamisole. *A. l.*, *Ascaris lumbricoides*; *T. t.*, *Trichuris trichiura*; Hw, hookworm. Prev, prevalence; PRR, prevalence risk ratio.

**Notes:**

^*^24 studies are included in meta-analysis of prevalence of STH after MDA, although three have low adherence rates (<70%) [32,36,39] and two have poor CR [13,28]. In some studies or cohort listed in Table 2, only integer value of rate or prevalence was available and could be abstracted.

^**^Time interval to first evaluation of treatment efficacy is given before the semicolon; subsequent follow-up intervals are listed after the semicolon.

^***^Efficacy of treatment was assessed 7-60 days posttreatment

^a^ Efficacy of treatment was assessed 7-60 days posttreatment and positives re-treated until cured. ^2T^Cure rate of second round of treatment.

^b^ Each child selected was negative for *A. lumbricoides* eggs on four consecutive stool examinations performed 2-4 weeks after treatment.

^ALB^ Efficacy of albendazole; ^MEB^ Efficacy of mebendazole.

^4R, 2R, 1R^ Repeated chemotherapy was carried out at intervals of 3, 6, or 12 months in 1 year.

^LA^ Low adherence rate, adherence rate during follow-up of this study or observational cohort was less than 70%.

^PC^ Poor cure rate, cure rate of *A. lumbricoides* was less than 50%.

GM = geometric mean, GM1 = geometric mean EPG including both positive and negative individuals, GM2 = geometric mean EPG excluding uninfected (only for the positive); AM = arithmetic mean, AM1 = arithmetic mean EPG including both positive and negative individuals, AM2 = arithmetic mean EPG excluding uninfected (only for the positive); II = (Re) infection intensity; IIR (infection intensity ratio) = intensity after treatment/intensity before treatment * 100%.
